# The Omics Revolution in Understanding Chicken Reproduction: A Comprehensive Review

**DOI:** 10.3390/cimb46060373

**Published:** 2024-06-20

**Authors:** Armughan Ahmed Wadood, Xiquan Zhang

**Affiliations:** 1 State Key Laboratory of Swine and Poultry Breeding Industry, Guangzhou 510642, China; armughanwadood@gmail.com; 2 Guangdong Provincial Key Lab of Agro-Animal Genomics and Molecular Breeding, and Key Lab of Chicken Genetics, Breeding and Reproduction, Ministry of Agriculture and Rural Affair, South China Agricultural University, Guangzhou 510642, China

**Keywords:** omics, proteomics, transcriptomics, metabolomics, chicken, reproduction

## Abstract

**Simple Summary:**

This review article discusses the impact of omics technologies on the understanding of chicken reproduction. Omics technologies such as genomics, transcriptomics, proteomics, and metabolomics have provided valuable insights into the molecular mechanisms underlying chicken reproduction. These technologies have allowed researchers to identify the key genes, proteins, and metabolites involved in reproductive processes, as well as their interactions. This article highlights the importance of omics approaches in advancing our understanding of chicken reproduction and their potential applications in improving reproductive efficiency and health in poultry production. Overall, this article emphasizes the transformative impact of omics technologies in unraveling the complexities of chicken reproduction.

**Abstract:**

Omics approaches have significantly contributed to our understanding of several aspects of chicken reproduction. This review paper gives an overview of the use of omics technologies such as genomics, transcriptomics, proteomics, and metabolomics to elucidate the mechanisms of chicken reproduction. Genomics has transformed the study of chicken reproduction by allowing the examination of the full genetic makeup of chickens, resulting in the discovery of genes associated with reproductive features and disorders. Transcriptomics has provided insights into the gene expression patterns and regulatory mechanisms involved in reproductive processes, allowing for a better knowledge of developmental stages and hormone regulation. Furthermore, proteomics has made it easier to identify and quantify the proteins involved in reproductive physiology to better understand the molecular mechanisms driving fertility, embryonic development, and egg quality. Metabolomics has emerged as a useful technique for understanding the metabolic pathways and biomarkers linked to reproductive performance, providing vital insights for enhancing breeding tactics and reproductive health. The integration of omics data has resulted in the identification of critical molecular pathways and biomarkers linked with chicken reproductive features, providing the opportunity for targeted genetic selection and improved reproductive management approaches. Furthermore, omics technologies have helped to create biomarkers for fertility and embryonic viability, providing the poultry sector with tools for effective breeding and reproductive health management. Finally, omics technologies have greatly improved our understanding of chicken reproduction by revealing the molecular complexities that underpin reproductive processes.

## 1. Introduction

**Figure 1:**
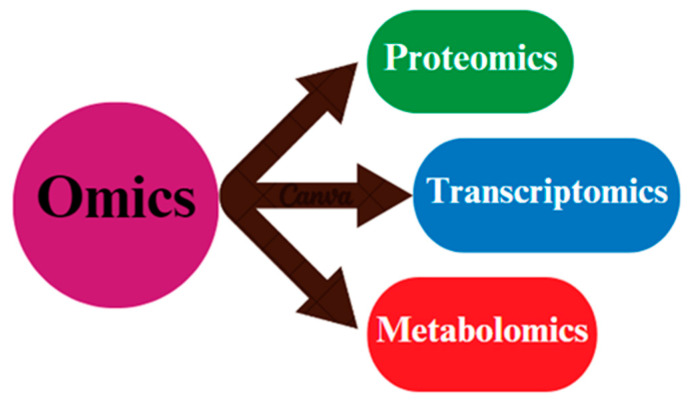
A schematic illusion diagram of the omics techniques (proteomics, transcriptomics, and metabolomics) that are commonly applied in biological samples.

Genomics, transcriptomics, proteomics, and metabolomics are all important approaches for better understanding chicken reproduction [1,2,3] (Figure 1). Genomic research has helped discover genes linked to reproductive features like fertility and egg production. Transcriptomics is used to understand the gene expression patterns at various phases of reproduction [4]. Proteomics research has provided insight into the proteins involved in reproductive processes, whereas metabolomics has assisted in discovering the metabolic pathways critical for fertility [5]. The integration of omics data has provided complete insights into the molecular mechanisms underpinning chicken reproduction, allowing for improved breeding methods and reproductive success in poultry [6].

Genomic research has helped to identify the genetic variants related to desirable reproductive features in chickens, such as egg quality, fertility, and broiler performance [7]. By studying the full genome, we can identify the genes associated with specific reproductive features, allowing for selective breeding to boost productivity and efficiency [8]. Single-cell sequencing methods have been used to evaluate gene expression patterns at the single-cell level, providing unparalleled resolution for investigating the variety of cell populations involved in chicken reproduction [9]. This method has revealed previously undiscovered cell types, regulatory networks, and dynamic changes throughout follicle development, embryogenesis, and gametogenesis.

Transcriptomic analysis enables the investigation of gene expression patterns across several stages of poultry reproduction. By analyzing the mRNA levels in reproductive organs or cells, we can identify the essential genes involved in follicle development, ovulation, sperm generation, and other reproductive processes [10]. This knowledge aids in understanding the regulatory networks that govern fertility and reproductive performance. Proteomics research has enhanced our understanding of the proteins involved in chicken reproduction and the intricate protein interactions that govern reproductive activities via the study of the entire set of proteins found in reproductive organs or cells [3,11]. Proteomic techniques have found biomarkers associated with fertility, egg quality, and sperm function, allowing for the development of diagnostic tools and breeding strategies [12].

Metabolomic analysis is concerned with investigating the entire range of small molecules (metabolites) found in biological samples and revealing metabolic pathways crucial for fertility, embryonic development, and reproductive health in chickens by characterizing the metabolites linked with reproductive activities. Metabolomics has revealed information on nutrient use, energy metabolism, and biomarkers in poultry reproduction [13,14].

Epigenomic research, concentrating on DNA methylation, histone modifications, and noncoding RNAs, has revealed the epigenetic regulation of gene expression in chicken reproductive organs [15,16]. We can learn about how environmental factors, stressors, and nutritional aspects affect reproductive outcomes in chickens by looking at epigenetic changes. Epigenomics has provided vital information about the heredity and plasticity of reproductive characteristics [17]. Metagenomic investigation of the chicken reproductive microbiome has arisen as a new area of study, providing insight into the relationships between host genetics, microbiota composition, and reproductive health. We can investigate the role of the microbiome in regulating fertility, disease resistance, and immunological responses in chickens by identifying the microbial populations in the reproductive tract [18]. Metagenomics has implications for developing techniques to modify the microbiome for better reproductive health [19].

Deciphering the intricate regulatory networks in chicken reproduction has been possible through the integration of several omics datasets, including those from transcriptomics, proteomics, metabolomics, and genomics [20]. Through the integration of several omics datasets, we can reveal the associations between genotype and phenotype, pinpoint pivotal biomarkers, and clarify the functional pathways that contribute to reproductive functioning [21,22]. A systems biology viewpoint on chicken reproduction is offered by multiomics integration, which provides a thorough grasp of the molecular pathways involved. Large-scale omics data in chicken reproductive studies may now be efficiently analyzed and interpreted because of developments in machine learning techniques and bioinformatics tools [23]. The identification of novel genetic markers, regulatory elements, and functional annotations pertinent to poultry reproduction has been expedited by bioinformatic pipelines for processing omics data, genome assembly, pathway analysis, and predictive modeling [24]. Additionally, phenotype–genotype correlations and predictions of complex features in chickens have been modeled using artificial intelligence techniques [25]. One of the most significant advantages of omics techniques is their ability to generate large volumes of biological data [26]. Integrating genomic, transcriptomic, proteomic, and metabolomic data provides a comprehensive picture of the molecular mechanisms that control chicken reproduction [2]. By merging multiomics datasets, we can find critical genetic regulators, signaling pathways, and metabolic networks that control reproductive features, providing a comprehensive picture of poultry genetics and reproduction. In conclusion, omics technologies have altered poultry genetics and reproductive research by enabling the investigation of the complicated molecular mechanisms that underpin these processes. The application of genomics, transcriptomics, proteomics, and metabolomics has improved our understanding of chicken reproduction, resulting in advances in breeding methods, disease management, and overall poultry production efficiency. Overall, recent advances in omics technology have improved our understanding of chicken reproduction by giving deep molecular insights, revealing novel biological insights, and hastening the discovery of genetic and regulatory variables that influence reproductive features in poultry [27].

### 1.1. Role of Proteomics in Chicken Reproduction

**Figure 2:**
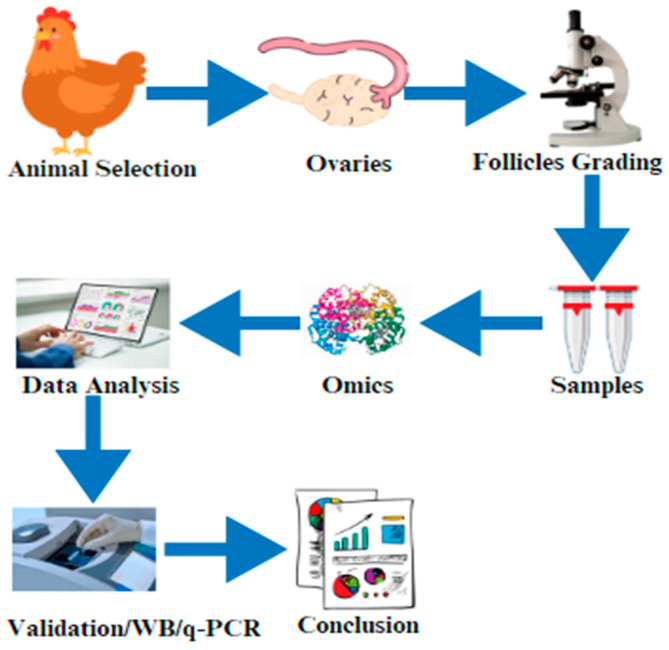
The graphical abstract of this current review article indicates the selection of the animals and the isolation of the ovaries. After that, ovary/follicle samples are prepared for omics (proteomics, transcriptomics, or metabolomics), data are validated by using Western blotting (WB)/real-time q-PCR, and the results are finally compiled.

Proteomics is the large-scale analysis of proteins and has emerged as a strong tool in biology, providing useful insights into a wide range of biological processes, including chicken reproduction [28]. The function of proteomics in chicken reproduction is an intriguing and difficult field of study that has the potential to transform our understanding of the mechanisms underpinning fertility, development, and the regulation of reproductive processes in these birds [29]. Proteomics, which can detect and quantify thousands of proteins at the same time, provides a complete perspective of the molecular alterations occurring in the chicken reproductive system [30] (Figure 2). One of the key applications of omics research in chicken reproduction is the identification of biomarkers that can be used as indicators of fertility or egg quality. Proteomics, which involves the study of the complete set of proteins in a cell, tissue, or organism, has particularly been instrumental in identifying protein biomarkers associated with chicken reproduction.

Proteomics has made major contributions to our understanding of chicken reproduction, particularly in the identification of the essential proteins involved in ovarian development and function [31]. The ovary is essential in female reproduction because it produces and releases eggs for fertilization [32]. Proteomic research on the chicken ovary has uncovered important proteins involved in follicular development, ovulation, and hormonal regulation, providing insight into the complex interplay of the molecular events that drive these processes [21,22,33]. Furthermore, proteomics has been useful in understanding the role of proteins in sperm production and function in male chickens. Sperm generation, maturation, and fertilization capacity are all important aspects of male fertility, and proteomic studies have discovered crucial proteins associated with these processes, providing vital insights into the molecular mechanisms underpinning successful rooster reproduction [34,35]. By analyzing the proteome of chicken reproductive tissues, researchers can identify specific proteins that are differentially expressed in fertile and infertile individuals, or in eggs of varying quality. These protein biomarkers can then be used to develop diagnostic tools that can accurately assess the fertility of breeding chickens or the quality of eggs produced. For example, a study may identify a specific protein that is abundantly expressed in the reproductive tract of fertile hens but is absent or expressed at lower levels in infertile hens. This protein biomarker can then be used in a diagnostic test to assess the fertility of individual hens or to predict the likelihood of successful fertilization.

In addition to studying reproductive organs, proteomics has been used to analyze seminal plasma, the fluid that surrounds and nourishes the sperm in the male reproductive tract [35]. Seminal plasma is a complex mixture of proteins that play important roles in sperm survival, motility, and fertilization [36]. Proteomic investigations of chicken seminal plasma have identified proteins that may act as indicators for male fertility and could be used to build novel reproductive technologies [37,38]. Furthermore, proteomics has been frequently utilized to explore how environmental factors such as nutrition, stress, and disease affect chicken reproduction [39]. Profiling the proteome of reproductive tissues under various conditions can identify the protein markers associated with poor reproductive performance, providing valuable insights into how external factors influence fertility and reproductive success in chickens [40]. The field of assisted reproductive technologies (ARTs), including artificial insemination and embryo modification, has also been affected by the application of proteomics in chicken reproduction [41]. The characterization of the proteome profiles of gametes, embryos, and reproductive organs can improve the success rates of assisted reproduction in chickens, refine ART procedures, and increase the effectiveness of fertility therapies [41]. Similarly, proteomics can also be used to identify the biomarkers associated with egg quality, such as proteins that are involved in the formation of the eggshell or proteins that are indicative of the nutritional quality of the egg. By developing diagnostic tools that utilize these protein biomarkers, farmers can assess the quality of the eggs produced by their chickens and make informed decisions on breeding strategies or nutritional interventions to improve egg quality.

Overall, proteomics has revolutionized our understanding of the molecular processes driving fertility, development, and reproductive function in these birds, which has fundamentally changed our understanding of chicken reproduction [42]. Proteome studies have cleared the path for cutting-edge research and useful applications in chicken breeding and production by revealing the complex interactions between the proteins involved in ovarian and sperm development, seminal plasma composition, and the effect of environmental factors on reproductive performance [43]. To summarize, the use of proteomics in chicken reproduction is a fast-emerging field with significant potential for improving our understanding of avian fertility and reproductive health [2].

#### 1.1.1. Role of Proteomics in Egg Production

Follicular formation in chickens is a complex biological phenomenon that is carefully controlled by a succession of molecular events [44,45]. The proteomic analysis enables the identification and quantification of the proteins involved in this process, revealing important insights into the underlying mechanisms [46]. By performing proteomic analysis on chicken samples, researchers can gain a better understanding of the molecular mechanisms involved in various physiological and pathological processes. This can help with identifying the key proteins that play a role in specific functions, such as growth and development, immune response, and disease progression. We can acquire a better grasp of the parameters influencing egg production by examining the protein content of chicken follicles at various developmental stages [47]. The capacity of proteomic analysis to find proteins that might act as indicators for reproductive success is one of the main benefits of using proteomics in poultry research [42]. Proteomic can also be used to identify particular proteins linked to higher egg productivity by comparing the protein profiles of hens that produce eggs well and those that do not. Proteomic studies can also provide insights into how different environmental conditions affect the development of chicken follicles and the quality of their eggs to find potential stress biomarkers and create plans to lessen their effects on egg production by examining the changes in follicles’ protein composition in response to various stressors, such as heat or nutrition [48]. Furthermore, proteomic analysis can help with identifying potential biomarkers for certain conditions or diseases, as well as with evaluating the effectiveness of treatments or interventions. Overall, proteomic analysis is a powerful tool that can provide important insights into the complex biological processes in chickens and other organisms.

Proteomics can be utilized not just to comprehend the molecular underpinnings of egg productivity but also to identify and track the presence of pollutants or diseases in chicken products [49], uncover proteins linked to threats to food safety, and create strategies for identifying and eliminating dangerous materials from the food supply chain by examining the protein profiles of eggs [50]. As such, the use of proteomics in poultry research could fundamentally alter our knowledge of the development of chicken follicles and egg output [51]. Proteomic analysis offers a comprehensive perspective on the protein landscape within chicken follicles [29], which offers new options for enhancing the reproductive efficiency in poultry farming and providing significant insights into the molecular mechanisms driving egg production [6]. In the context of enhancing reproductive efficiency and driving egg production in chickens, proteomics can be used to identify key proteins and pathways involved in processes such as ovulation, fertilization, and egg formation. By conducting proteomic analysis on reproductive tissues, fluids, or cells of chickens, researchers can gain insights into the molecular mechanisms underlying these processes. This information can then be used to develop strategies to enhance reproductive efficiency and increase egg production in chickens. For example, proteomic analysis may reveal the proteins that are crucial for oocyte maturation, sperm function, or hormone regulation. By targeting these proteins with specific interventions, such as nutritional supplements or gene editing techniques, researchers may be able to improve the reproductive performance of chickens.

#### 1.1.2. Proteomic Techniques Used in Chickens

Proteomics is an essential tool for comprehending the molecular mechanisms underpinning a variety of physiological activities in chickens, including health, illness resistance, and growth performance [29]. Two-dimensional gel electrophoresis is a technique used to separate proteins based on their isoelectric point and molecular weight. It provides high resolution but can be time consuming and have low reproducibility. Liquid chromatography–tandem mass spectrometry (LC-MS) is a powerful tool used for protein identification and quantification that has high sensitivity and accuracy. It can be used in conjunction with 2-D gel electrophoresis to overcome its limitations. Two-dimensional gel electrophoresis (2-DE) is one of the most popular proteomic methods used in chicken research [52,53]. By dividing proteins according to their molecular weight and isoelectric point (pI), this technique offers a thorough understanding of the chicken proteome. Through the process of comparing the protein profiles between distinct situations, such as ill versus healthy birds or treated versus untreated groups, the proteins that exhibit differential expression can be discerned, potentially having an impact on the phenotypes that are seen [53,54].

Matrix-assisted laser desorption/ionization (MALDI) is a mass spectrometry technique that is often used for protein profiling and identification. It has a fast analysis speed and high sensitivity, but it may not be as effective as LC-MS in terms of data quality and proteome coverage. MALDI makes it possible to identify and measure proteins in complicated biological samples [55]. Liquid chromatography–mass spectrometry (LC-MS) and matrix-assisted laser desorption/ionization (MALDI) are two popular forms of mass spectrometry (MS) [56]. Because of the excellent sensitivity and specificity of these methods, we can even identify proteins with low abundances and post-translational modifications [57].

**Figure 3:**
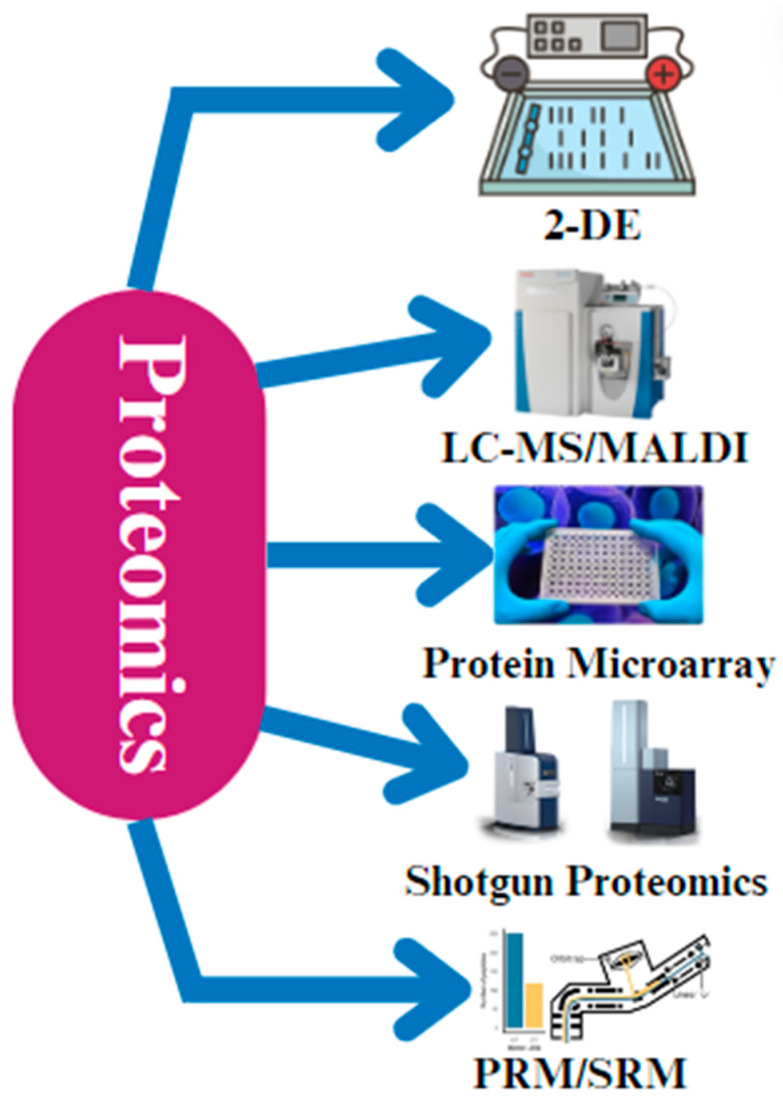
The different techniques that are applied during proteomic analysis. The most common techniques that are used are 2-D gel electrophoresis, liquid chromatography–mass spectrometry (LC-MS), matrix-assisted laser desorption/ionization (MALDI), protein microarray technology, shotgun proteomics, parallel reaction monitoring (PRM), and selective reaction monitoring (SRM).

Apart from 2-DE and MS, protein microarray technology has become an important tool in the field of chicken proteomics [58] (Figure 3). Protein microarray technology is a high-throughput method for studying protein interactions and functions. It allows for the simultaneous analysis of thousands of proteins but may have limited sensitivity and specificity compared to mass-spectrometry-based methods. Protein interactions with DNA, other proteins, and small molecules can be studied in high-throughput using protein microarrays, enabling systematic analysis of the roles and interactions of thousands of chicken proteins by organizing them on a stable substrate [58]. Furthermore, the field of chicken research has seen a rise in the use of shotgun proteomics, which breaks down proteins into peptides and analyzes them using mass spectrometry. Shotgun proteomics is a mass-spectrometry-based approach that involves digesting proteins into peptides and analyzing them in a high-throughput manner. It provides comprehensive proteome coverage but may have limitations in terms of quantifying proteins and identifying low-abundance proteins. This method is especially helpful for large-scale research that attempts to thoroughly characterize the chicken proteome since it makes a high number of proteins identifiable in a single experiment [59]. Recent developments in targeted proteomic methods, such as parallel reaction monitoring (PRM) and selective reaction monitoring (SRM), have made it possible to measure particular proteins very precisely and sensitively [60,61]. They offer high sensitivity and specificity but are more labor-intensive compared to shotgun proteomics. These methods yield important information, particularly when examining important regulatory proteins or biomarkers in chickens [59,62].

### 1.2. Role of Transcriptomics in Chicken Reproduction

Transcriptomics, or the study of an organism’s entire set of RNA transcripts, is an important technique used for understanding numerous biological processes, including chicken reproduction [63]. Transcriptomics analysis can provide valuable insights into the molecular mechanisms driving fertility, embryo development, and overall reproductive success by studying the gene expression profiles of chickens during critical reproductive stages [64].

Transcriptomics is important in investigating chicken reproduction because it can uncover the critical genes and pathways involved in reproductive activities and identify genes that are up- or downregulated during critical events such as ovulation, fertilization, and embryonic development by comparing gene expression levels across distinct reproductive states [65]. Such studies might be useful in understanding the molecular basis of reproductive failure and creating fertility-enhancing techniques in chicken breeding programs [66]. Transcriptomics can also shed light on the effect of external factors such as nutrition, stress, and environment on chicken reproductive health to discover the biological mechanisms by which these factors influence reproductive outcomes by examining changes in gene expression in response to them [67].

Transcriptomic studies in chicken reproduction have also helped to identify molecular markers linked to key reproductive features and to uncover genetic markers that can be employed in selective breeding by comparing gene expression patterns to phenotypic features such as egg production, hatchability, and sperm quality [68,69]. Furthermore, transcriptomics is critical for understanding epigenetic control in chicken reproduction. Epigenetic changes, such as DNA methylation and histone modifications, can affect gene expression patterns without changing the underlying DNA sequence [70]. Transcriptomic analysis enables us to understand how these epigenetic mechanisms control gene expression during critical reproductive events and how they might be targeted to improve chicken reproductive outcomes [71].

Transcriptomic investigations can assist veterinarians and breeders in early diagnosis and the implementation of focused treatment methods by identifying the biomarkers linked to reproductive diseases or inadequate fertility [72]. Transcriptomic research, by deciphering the complex gene expression profiles associated with critical reproductive processes, provides the path for the identification of genetic markers, breeding program optimization, and improved reproductive outcomes in poultry production [13,73].

#### Techniques Used for Chicken Transcriptomics

**Figure 4:**
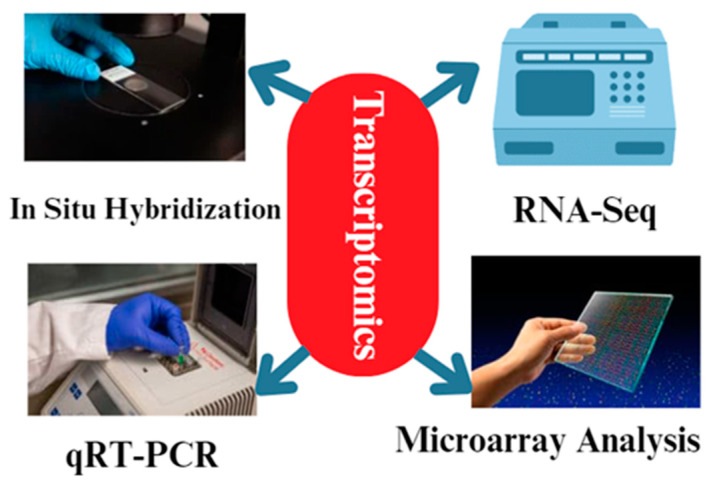
The different methodologies used during transcriptomics analysis. The most common techniques are qRT-PCR and RNA-Seq. In addition, microarray analysis and in situ hybridization techniques are used for transcriptomics analysis.

Various methodologies are employed in transcriptomics to examine the patterns of gene expression in animal reproduction [74] (Figure 4). These methodologies enable us to discern the genes that are expressed differently, as well as the signaling pathways and regulatory networks that play a role in reproductive processes [74]. The common techniques used in transcriptomics for animal reproduction include RNA sequencing (RNA-Seq), microarray analysis [75], quantitative real-time polymerase chain reaction (qRT-PCR) [76], and in situ hybridization [77]. RNA sequencing, also known as RNA-Seq, is a very efficient method that enables the sequencing and measuring of the abundance of all RNA molecules present in a given sample. RNA-Seq is a technique that can be used to gather data on gene expression levels, identify alternative splicing processes, and uncover new transcripts [78]. Through the comparison of transcriptomes from various reproductive organs or developmental stages, we can discern the genes that exhibit unique expression in reproductive cells and tissues [79]. RNA-Seq techniques are used in chicken reproduction research to study the gene expression patterns in reproductive tissues and unravel the molecular mechanisms underlying various reproductive processes. In particular, the genes RAC1, MRE11A, MAP7, and SOX5 have been identified as playing important roles in chicken reproduction based on RNA-seq studies, which uncovered differentially expressed genes in the ovaries, testes, and reproductive tracts of male and female chickens at distinct developmental stages [80].

Microarray analysis is a commonly employed method in transcriptomics that enables the evaluation of the expression levels of several genes simultaneously in a single experiment [81]. Microarrays are composed of many DNA probes that bind to RNA molecules in a sample, yielding data on the levels of gene expression [82]. Microarray analysis was employed to examine the gene expression patterns in reproductive tissues, including the ovary, testis, and uterus [83]. It was also utilized to identify the genes that exhibit differential expression during reproductive processes [84]. Microarray analysis is a prominent transcriptomics approach [74]. Microarrays have enabled the concurrent evaluation of the expression levels of thousands of genes in a sample and the uncovering of the essential genes and pathways involved in chicken reproduction by comparing gene expression profiles across reproductive tissues or healthy and sick hens [85,86].

In addition to RNA-Seq and microarray analysis, quantitative real-time polymerase chain reaction (qRT-PCR) is a popular technique in transcriptomics [87]. With qRT-PCR, we can estimate the expression levels of individual genes with high sensitivity and accuracy [88]. This technique is frequently used to validate the results of RNA-Seq or microarray research, as well as to investigate the dynamics of gene expression at various phases of chicken reproduction [89]. Quantitative real-time polymerase chain reaction (qRT-PCR) is a very accurate and precise method frequently employed to confirm the gene expression data acquired from RNA-Seq or microarray research [90]. Quantitative reverse-transcription polymerase chain reaction (qRT-PCR) enables the calculation of the gene expression levels in a sample and confirms the differential expression of certain genes of interest. qRT-PCR is commonly employed to confirm the candidate genes identified in transcriptome investigations and to assess the expression patterns of genes implicated in reproductive functions [91].

Currently, single-cell RNA sequencing (scRNA-seq) has been developed as a strong transcriptomics method [92]. This technique enables the study of gene expression patterns at the individual cell level, resulting in a more complete understanding of the heterogeneity within complex tissues such as the chicken reproductive system. By analyzing the transcriptomes of individual germ cells, follicle cells, and embryonic cells, we can discover the regulatory networks and cell-to-cell communication routes involved in chicken reproduction [93]. Overall, transcriptomics has transformed our understanding of the molecular pathways that drive chicken reproduction and can be used to understand the complexity of gene expression in chickens by using modern techniques such as RNA sequencing, microarray analysis, qRT-PCR, and single-cell RNA sequencing [94]. These findings not only have expanded our understanding of avian reproduction but also have implications for increasing chicken production and fertility management tactics in the poultry business.

In situ hybridization is a method used to observe the precise location of particular RNA molecules within cells and tissues [95]. In situ hybridization employs tagged RNA probes that bind to the complementary RNA molecules in a tissue segment, enabling observation of the expression patterns of particular genes [96]. In situ hybridization has been utilized to investigate the expression patterns of the genes implicated in reproductive processes such as folliculogenesis, spermatogenesis, and embryo implantation [97].

### 1.3. Role of Metabolomics in Chicken Reproduction

Metabolomics is an advanced scientific method that is used to thoroughly analyze the small-molecule metabolites present in biological systems [98]. Through the analysis of metabolite profiles, useful knowledge of the metabolic pathways, biochemical processes, and physiological functions that support different biological functions can be described [99]. Metabolomics is a valuable technique in the study of chicken reproduction since it allows for the investigation of the metabolic changes that take place during several reproductive phases, including follicular development, ovulation, fertilization, and embryogenesis [100].

Metabolomics has been used to investigate the relationship between chicken reproduction efficiency and metabolic changes, yielding important insights into the factors that affect reproductive outcomes [24] and detecting distinct metabolic indicators linked to fertility, fecundity, and hatchability in chickens by examining the metabolite profiles of various reproductive organs, fluids, and biofluids [101]. Metabolomics is essential for comprehending the influence of many environmental conditions, genetic factors, and dietary interventions on the efficiency of chicken reproduction [102]. Through the comparison of metabolite profiles in hens exposed to various environments, we can detect the metabolic signatures linked to enhanced reproductive success [103] and can be used to monitor the reproductive health of chickens, including hormone levels and fertility. This can help identify and address any potential issues that may affect breeding success.

Moreover, metabolomics enables the detection of possible biomarkers associated with fertility-related illnesses and reproductive diseases in chickens [1,104]. Through the examination of metabolic disruptions linked to illnesses including ovarian dysfunction, reproductive tract infections, and hormonal abnormalities, diagnostic instruments can be developed to identify and track these problems at an early stage [105]. By taking a proactive approach, timely interventions can be implemented to manage reproductive health issues in hens. This helps to minimize economic losses and promote animal welfare. Furthermore, it can be used to analyze the nutritional content of chicken feed and optimize the diet for optimal growth and reproductive performance. This can help improve the overall productivity of the chicken population.

#### Techniques Applied in Metabolomics

**Figure 5:**
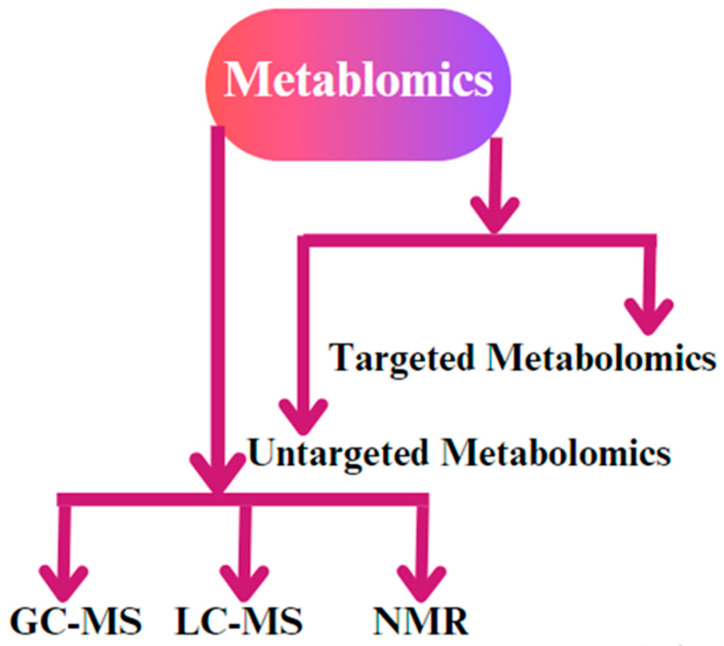
The different types of metabolomics techniques used for biological sample analysis. The most common methodologies that are applied are gas chromatography–mass spectrometry (GC-MS), liquid chromatography–mass spectrometry (LC-MS), and nuclear magnetic resonance (NMR) spectroscopy. In addition, untargeted metabolomic and targeted metabolomic techniques are used for biological replicates.

Gas chromatography–mass spectrometry (GC-MS) is a commonly employed method in metabolomics that enables the isolation and identification of metabolites by analyzing their mass and chemical characteristics [106]. It involves separating and analyzing compounds based on their volatility and mass-to-charge ratio, so is ideal for analyzing small volatile molecules to identify compounds that are thermo-stable and can be vaporized without decomposition. GC-MS can be employed to examine the metabolic profiles of reproductive tissues, such as the ovary and oviduct [107]. Through the comparison of metabolite profiles throughout several reproductive phases, scientists can pinpoint the crucial metabolites linked to activities such as ovulation and fertilization [108] (Figure 5).

Liquid chromatography–mass spectrometry (LC-MS) is a highly effective technique used in metabolomics to accurately detect and measure the concentration of metabolites in intricate biological samples [109]. It involves separating and analyzing compounds based on their polarity and mass-to-charge ratio, so is ideal for analyzing the polar compounds and large molecules for analyzing compounds that are not volatile or thermo-stable. LC-MS is an essential tool in the examination of chicken reproduction as it enables the analysis of the metabolic alterations that take place throughout embryonic development [110]. Through the analysis of the metabolites in embryonic tissue samples at various stages of development, scientists can reveal the essential metabolic pathways that are crucial for appropriate growth and differentiation [111].

Nuclear magnetic resonance (NMR) spectroscopy is a noninvasive method that offers comprehensive insights into the chemical composition of metabolites [112]. It involves analyzing the chemical structure of compounds based on the interactions between nuclei and magnetic fields and provides information on the chemical environment and the connectivity of atoms in a molecule. NMR spectroscopy is a useful tool for examining the metabolic alterations in reproductive fluids, including blood plasma and follicular fluid [113]. Through the examination of the NMR spectra of these fluids, scientists can detect biomarkers linked to reproductive health and fertility [114].

Untargeted metabolomics is a comprehensive method that seeks to identify a wide range of metabolites in a biological sample without any prior information about their chemical identification [115], and it can be used to discover novel biomarkers or metabolic pathways related to chicken reproduction. In the study of chicken reproduction, untargeted metabolomics has provided a broad overview of the metabolic pathways involved in key reproductive processes [14,116]. In contrast to untargeted metabolomics, targeted metabolomics focuses on the quantification of specific metabolites or metabolic pathways of interest [117]. Targeted metabolomics is a useful technique in chicken reproduction research for quantifying the amounts of crucial metabolites involved in reproductive processes, including hormone synthesis and embryonic growth. Researchers might enhance their comprehension of the metabolic changes that take place during crucial stages of chicken reproduction by focusing on particular metabolites [118].

In summary, metabolomics approaches have greatly transformed the investigation of chicken reproduction by offering a thorough examination of the metabolic alterations that take place throughout crucial reproductive processes. Gas chromatography–mass spectrometry and nuclear magnetic resonance spectroscopy are two techniques that offer distinct advantages in the realm of metabolomics research.

### 1.4. Genomics/Epigenomics and Chicken Reproduction

Genomics is the scientific discipline that investigates the complete genetic material of an organism, encompassing its DNA sequence [119]. Genomics has facilitated the identification of crucial genes and genetic markers linked to qualities such as fertility, hatchability, and egg output in the field of chicken reproduction [120]. Through the examination of the chicken genome, we can identify specific genetic changes that have an impact on reproductive performance [121].

A notable progress in genomics is the utilization of genome-wide association studies (GWAS) to chart the genetic markers associated with reproductive characteristics in chickens [122,123]. GWAS have revealed genes and genomic areas that are crucial in reproductive functions, such as hormone control, follicle growth, and sperm quality [122,124]. Genomic data can be utilized by breeders to make well-informed choices regarding mating pairs, thereby maximizing genetic variety and enhancing the overall reproductive efficacy of chicken populations [125].

Epigenomics is the field of study that specifically examines the modifications in gene expression that are not a result of changes in the DNA sequence [126]. Epigenomics is essential in controlling gene expression during important stages of development and reproduction in chickens [127]. Epigenetic alterations, such as DNA methylation and histone modification, can influence reproductive processes in chickens [15,128]. Epigenetic modifications in the genes associated with sex determination and embryo development can impact the rates of fertility and hatchability, as an example [129]. Studying the epigenetic mechanisms involved in chicken reproduction has the potential to generate novel approaches for enhancing reproductive efficiency [130]. Epigenomics can impact gene expression without modifying the DNA sequence, which is one of its main advantages. Epigenetic control enables the modulation of gene activity in hens’ reproductive processes in response to environmental cues, facilitating precise adjustments [131].

#### Integration of Genomics/Epigenomics via Omics

Omics techniques, such as genomics and epigenomics, allow for the comprehensive analysis of the chicken genome and epigenome [132]. These techniques involve high-throughput sequencing and bioinformatics analysis and generate large-scale datasets that can provide a detailed picture of the genetic and epigenetic factors influencing chicken reproduction [133]. By integrating these omics approaches, we can identify the key genes and pathways that are involved in reproductive processes and gain a better understanding of the complex interactions between genetics and the environment [134].

One of the main advantages of integrating genomics and epigenomics using omics in chicken reproduction is the ability to identify novel biomarkers for reproductive traits [15]. By analyzing the genetic and epigenetic signatures associated with desirable reproductive traits, researchers can develop molecular markers that can be used for breeding selection [135]. This can help poultry breeders to more effectively select for desirable traits, such as increased egg production and fertility, leading to improved breeding programs and overall productivity.

Additionally, the integration of genomics and epigenomics in chicken reproduction can provide valuable insights into the molecular mechanisms underlying reproductive disorders and diseases [15]. By studying the genetic and epigenetic factors associated with reproductive disorders, researchers can identify potential therapeutic targets and develop targeted interventions to improve reproductive health in chickens [136]. In conclusion, the integration of genomics and epigenomics through omics techniques in chicken reproduction holds great promise for advancing our understanding of the molecular mechanisms underlying important reproductive processes in chickens [137].

## 2. Overall Conclusions

The incorporation of omics in investigating chicken reproduction has greatly enhanced our comprehension of the molecular pathways that regulate reproductive efficiency, utilizing the genomes, transcriptomics, metabolomics, and proteomics data integration to pinpoint the crucial genes, pathways, and biomarkers linked to fertility, egg production, and other reproductive features. Omics techniques can be utilized to formulate precise breeding tactics, enhance reproductive results, and optimize the overall efficiency of chicken farming. Overall, omics techniques have demonstrated their effectiveness in improving chicken reproductive efficiency. We can utilize the knowledge acquired from genomic, transcriptomic, metabolomic, and proteomic investigations to make well-informed choices that enhance breeding programs and reproductive performance in chicken populations. By integrating data from these different omics approaches, researchers can create a more comprehensive and detailed picture of the molecular mechanisms that underlie chicken reproduction. This holistic approach can help identify potential biomarkers for reproductive health and fertility, as well as inform breeding strategies to improve reproductive performance in chicken populations. This knowledge holds great potential for improving the efficiency and sustainability of poultry farming in the future.

## 3. Recommendations

Omics technologies have the potential to aid researchers and breeders in comprehending the genetic foundation of reproductive features, detecting biomarkers for fertility and egg production, and refining breeding tactics to improve overall reproductive efficiency in chickens. An important suggestion for the use of omics techniques in chicken reproduction efficiency is the use of genetic information to enhance breeding strategies. Genomic technologies, such as next-generation sequencing and genotyping arrays, have greatly enhanced our capacity to detect the genetic changes linked to significant reproductive characteristics in chickens. Through the implementation of genome-wide association studies (GWAS) and genomic selection, breeders can identify specific genes and genomic regions that have an impact on fertility, hatchability, and other reproductive features. Researchers can obtain useful insights into the genetic and molecular pathways that control reproductive qualities in chickens by utilizing genomics, transcriptomics, proteomics, and metabolomics.

## 4. Future Prospectives

Omics techniques, such as genomics, transcriptomics, proteomics, and metabolomics, have fundamentally transformed our approach to studying biological systems. These technologies have the potential to significantly improve our comprehension of the fundamental mechanisms that control fertility, hatchability, and overall reproductive efficiency in poultry, specifically in the realm of chicken reproduction. Through the implementation of genomic studies on diverse chicken breeds exhibiting differing levels of reproductive efficiency, we can precisely identify the genetic variants that are associated with specific attributes, including egg production, embryo development, and sperm quality. Through transcriptomic analysis, we can examine gene expression profiles in fertile and infertile chickens and pinpoint crucial genes and pathways linked to successful reproduction. Proteomics, which investigates the complete collection of proteins generated by an organism, can be beneficial in comprehending the mechanisms that contribute to the effectiveness of chicken reproduction. Through the examination of the protein makeup of reproductive organs such as the ovary, oviduct, and sperm, we can pinpoint crucial proteins that play a role in significant reproductive functions such as egg production, fertilization, and embryo growth.

Metabolomics, a field that examines tiny molecules like metabolites and hormones, might offer vital insights into the metabolic pathways that regulate reproductive activities in chickens. Through the examination of the metabolic profiles of chickens that are capable of reproducing and those that are not, we can detect crucial metabolites that are linked to the effectiveness of reproduction. Epigenetic modifications have been shown to play a crucial role in reproductive processes. Omics techniques, such as epigenomics, can be used to study the epigenetic changes that occur during different stages of the reproductive cycle in chickens.

Omics techniques can be used to develop personalized reproductive management strategies for chickens. By understanding the genetic and molecular profiles of individual birds, it is possible to tailor management practices to optimize reproductive performance. This can lead to improved efficiency and sustainability in the poultry industry. Omics techniques can be used to identify biomarkers of reproductive diseases and disorders in chickens. This can aid in the early diagnosis and treatment of these conditions, leading to improved reproductive health and productivity in poultry flocks. The use of omics techniques, such as gene editing, can allow for the manipulation of specific genes involved in reproductive processes, which can lead to the development of chickens with improved reproductive performance and disease resistance. In order to improve disease resistance and reproductive performance in chickens, omics techniques, such as gene editing, can be utilized to specifically target and manipulate genes involved in reproductive processes. One way gene editing can be used is through the CRISPR/Cas9 system, which allows for the precise editing of genes by introducing specific mutations or deletions. By targeting the genes involved in immune response or reproductive processes, it is possible to enhance disease resistance and reproductive performance in chickens. For example, the genes involved in immune response can be edited to increase the chicken’s ability to fight off pathogens and improve overall health. Additionally, the genes involved in reproductive processes, such as fertility and egg production, can be targeted to enhance reproductive performance in chickens. By using omics techniques like gene editing, researchers can selectively manipulate specific genes to improve disease resistance and reproductive performance in chickens. This can lead to healthier and more productive poultry populations, ultimately benefiting both producers and consumers.

By integrating omics data with bioinformatics, researchers can analyze and interpret large datasets to identify the key genes, pathways, and biological processes involved in chicken reproduction. Bioinformatics tools and algorithms help in the identification of the biomarkers, gene regulatory networks, and signaling pathways that are critical for reproductive processes. Systems biology takes the integration of omics data a step further by incorporating computational modeling and simulation to understand the complex interactions and dynamics of biological systems. By modeling the regulatory networks and pathways involved in chicken reproduction, researchers can gain insights into the underlying mechanisms and predict the outcomes of genetic or environmental perturbations. Overall, the integration of omics with bioinformatics and systems biology has revolutionized our understanding of chicken reproduction by providing a comprehensive view of the molecular mechanisms underlying this critical biological process. This interdisciplinary approach has the potential to drive future advancements in poultry breeding and production, ultimately leading to improved efficiency and sustainability in the poultry industry.

The role of omics techniques in chicken reproduction is constantly evolving and expanding. These techniques have already provided valuable insights into the genetic and molecular mechanisms involved in reproductive processes. With further advancements in technology and research, omics techniques have the potential to revolutionize the way we manage and improve reproductive performance in chickens. They can also aid in the development of novel treatments for reproductive disorders, leading to improved productivity and sustainability in the poultry industry. Therefore, it is essential to continue investing in research and development in this field to fully realize the potential of omics techniques in chicken reproduction.
